# Decreased DNA methylation at promoters and gene-specific neuronal hypermethylation in the prefrontal cortex of patients with bipolar disorder

**DOI:** 10.1038/s41380-021-01079-0

**Published:** 2021-04-20

**Authors:** Miki Bundo, Junko Ueda, Yutaka Nakachi, Kiyoto Kasai, Tadafumi Kato, Kazuya Iwamoto

**Affiliations:** 1 Department of Molecular Brain Science, Graduate School of Medical Sciences, Kumamoto University, Kumamoto, Japan; 2 Laboratory for Molecular Dynamics of Mental Disorders, RIKEN Center for Brain Science, Saitama, Japan; 3 Department of Neuropsychiatry, Graduate School of Medicine, The University of Tokyo, Tokyo, Japan; 4 The International Research Center for Neurointelligence (WPI-IRCN) at The University of Tokyo Institutes for Advanced Study (UTIAS), Tokyo, Japan; 5 Department of Psychiatry and Behavioral Science, Graduate School of Medicine, Juntendo University, Tokyo, Japan

**Keywords:** Bipolar disorder, Genetics

## Abstract

Bipolar disorder (BD) is a severe mental disorder characterized by repeated mood swings. Although genetic factors are collectively associated with the etiology of BD, the underlying molecular mechanisms, particularly how environmental factors affect the brain, remain largely unknown. We performed promoter-wide DNA methylation analysis of neuronal and nonneuronal nuclei in the prefrontal cortex of patients with BD (*N* = 34) and controls (*N* = 35). We found decreased DNA methylation at promoters in both cell types in the BD patients. Gene Ontology (GO) analysis of differentially methylated region (DMR)-associated genes revealed enrichment of molecular motor-related genes in neurons, chemokines in both cell types, and ion channel- and transporter-related genes in nonneurons. Detailed GO analysis further revealed that growth cone- and dendrite-related genes, including *NTRK2* and *GRIN1*, were hypermethylated in neurons of BD patients. To assess the effect of medication, neuroblastoma cells were cultured under therapeutic concentrations of three mood stabilizers. We observed that up to 37.9% of DMRs detected in BD overlapped with mood stabilizer-induced DMRs. Interestingly, mood stabilizer-induced DMRs showed the opposite direction of changes in DMRs, suggesting the therapeutic effects of mood stabilizers. Among the DMRs, 12 overlapped with loci identified in a genome-wide association study (GWAS) of BD. We also found significant enrichment of neuronal DMRs in the loci reported in another GWAS of BD. Finally, we performed qPCR of DNA methylation-related genes and found that *DNMT3B* was overexpressed in BD. The cell-type-specific DMRs identified in this study will be useful for understanding the pathophysiology of BD.

## Introduction

Bipolar disorder (BD), also known as manic depressive illness, is a severe and common mental disorder characterized by repeated mood swings of depressive and manic episodes, with elevated rates of mortality [1, 2]. Early epidemiological and linkage studies suggested that BD is a highly heritable disorder caused by a complex interaction of genetic and environmental risk factors [3]. Genome-wide association studies (GWAS) revealed that BD is a polygenic disorder caused by multiple genetic risks with small effect sizes, similar to schizophrenia (SZ), and shared genetic risks with other psychiatric disorders, such as SZ and autism [4–6]. Although genetic landscape of BD is gradually becoming understood, heritability estimated from epidemiological studies is modestly accounted for by the genetic studies.

Epigenetics, including DNA methylation, reflects gene-environment interactions during development and affects long-lasting gene expression status [7]. Therefore, unraveling the epigenetic landscape of psychiatric disorders will contribute to the understanding of the heritability and pathophysiology of psychiatric disorders [8–11]. In BD, several candidate-gene-based approaches have been performed, such as *BDNF, COMT*, and *SLC6A4* genes in postmortem brains [9]. In addition, comprehensive DNA methylation studies have revealed expression-linked DNA methylation changes in the cerebellum [12], accelerated aging in the hippocampus [13], loss of brain laterality associated with *TGFB2* methylation [14], and methylation imbalance of synaptic function-related genes between the frontal and temporal cortices [15]. However, there have been no established findings that were replicated in multiple studies.

DNA methylation status in brain cells shows great variation among cell types [16–18]. Therefore, cell-type-specific epigenetic analysis will also be important. Recent studies have highlighted that cell-type-specific epigenetic differences are linked to SZ and neuropsychiatric traits [19–22]. In BD, hypomethylation of the *IGF2* enhancer, which is associated with increased tyrosine hydroxylase protein levels, has been reported in isolated neuronal nuclei [23].

In this study, we performed promoter-wide DNA methylation analysis of neuronal and nonneuronal nuclei in the prefrontal cortex (PFC) of patients with BD. In addition to identifying cell-type-specific differentially methylated regions (DMRs), we found hypomethylation at promoters in both cell types in BD patients. The affected genes included hypomethylation of molecular motor-related genes in neurons, chemokine-related genes in both cell types, and ion channel- and transporter-related genes in nonneurons. We also found neuron-specific hypermethylation of growth cone- and dendrite-related genes. We then assessed the effect of medication by using neuroblastoma cells and found that up to 37.9% of DMRs in BD patients overlapped with mood stabilizer-induced DMRs. Interestingly, mood stabilizer-induced DMRs showed the opposite direction of changes in DMRs in BD, suggesting the therapeutic effects of mood stabilizers on DNA methylation. Among the DMRs, 12 overlapped with loci identified by a GWAS of BD [6]. We found significant enrichment of neuronal DMRs in the loci reported in another GWAS of BD [24]. We also found overexpression of *DNMT3B* in BD and SZ, suggesting possible molecular mechanisms of neuronal hypermethylation.

## Materials and methods

Details of methods including cell culture, data analysis, reduced representation bisulfite sequencing (RRBS), and qPCR were described in Supplementary Methods.

### Postmortem brains

PFC (Brodmann area 46) samples of patients with BD (*N* = 34) and controls (*N* = 35) were obtained from the Stanley Medical Research Institute (Table S1). This study was approved by the ethics committees of the participating institutes (the Research Ethics Committee of Kumamoto University, the Research Ethics Committee of the Faculty of Medicine of The University of Tokyo, the Ethical Review Board of Juntendo University, and the Wako 1st Research Ethics Committee of RIKEN).

### Nuclei preparation

Neuronal and nonneuronal nuclei fractions were separated by NeuN-based cell sorting [16]. In brief, after homogenization of fresh-frozen brain samples, the nuclear fraction was retrieved by Percoll discontinuous density gradient centrifugation. An anti-NeuN antibody (#MAB377, Millipore, Burlington, MA, USA) conjugated with Alexa Fluor 488 was used for staining. NeuN+ and NeuN− nuclei were sorted using the FACS Aria system (BD Biosciences, Franklin Lakes, NJ, USA) as previously described [16].

### Enrichment of methylated DNA and tiling arrays

Enrichment of methylated DNA was performed using MethylCollector (Active Motif, Carlsbad, CA, USA) according to the manufacturer’s protocol. A total of 100 ng of DNA was used. Methylated DNA was retrieved in 100 µL of elution buffer. In qPCR, aliquots of eluted methylated DNA were used for quantification. Probe preparation and labeling for Affymetrix human promoter 1.0R tiling arrays were performed according to the Affymetrix chromatin immunoprecipitation assay protocol (Affymetrix, Santa Clara, CA, USA). The array covers 25,500 human promoters by 4.6 million 25-mer oligo-probes. Each promoter covers ~7.5 kb upstream through 2.5 kb downstream of the transcription start site by 35 bp probe spacing. In the postmortem brain experiment, all experiments were performed in duplicate using independently prepared probes (experiments 1 and 2). The total numbers of array data points were therefore 136 and 140 for BD patients and controls, respectively. References were prepared by applying human genomic DNA amplified by a GenomiPhi V2 DNA amplification kit (GE Healthcare, Chicago, IL, USA) to MethylCollector. The number of methylated regions (MRs) of each sample was counted by MAT [25] using two replicate sample datasets (experiments 1 and 2) as one target group and a reference dataset (whole-genome amplified samples) as a reference group. DMRs were independently identified in experiments 1 and 2 by comparing the patient and control datasets. The DMRs detected in experiments 1 and 2 were then intersected by bedtools and used for further analysis.

### Analysis of hmC

To identify hydroxymethylated regions (HMRs), genomic DNA extracted from neuronal and nonneuronal nuclei isolated from two control subjects was used for immunoprecipitation using rat 5-hydroxymethylcytosine (5-hmC) monoclonal antibody included in the hMeDIP kit (#AF-104-0016) according to the manufacturer’s protocol (Diagenode, Denville, NJ, USA). The reference was prepared by performing immunoprecipitation using rat IgG antibody. Probe preparation and tiling array analysis were performed as described above.

## Results

### Promoter hypomethylation of the PFC in patients with BD

**Fig. 1:**
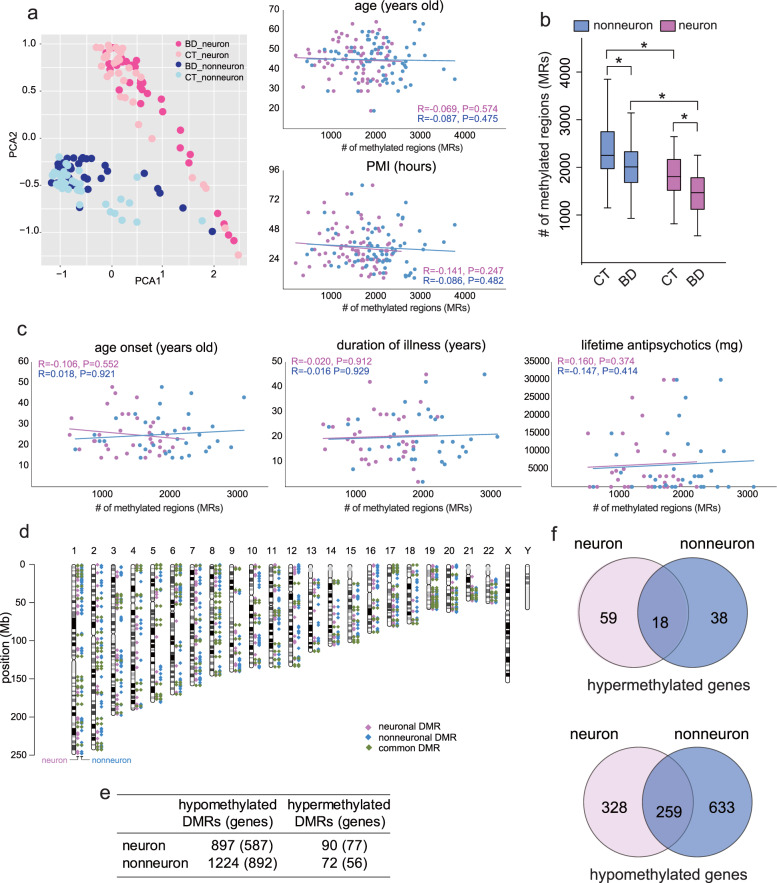
MRs and DMRs in BD. **a** PCA of the MRs of each sample and the effect of age and PMI. Spearman’s rank correlation coefficient and *P* value are given. Pink and blue colors indicate neurons and nonneurons, respectively. **b** Decreased number of MRs in BD. An asterisk (*) shows significant changes by the Mann–Whitney test. **c** Effect of age onset, duration of illness, and lifetime antipsychotics on MRs in BD. Lifetime antipsychotics are given in fluphenazine equivalents. One patient with excess lifetime antipsychotics was removed from the plot. The presence or absence of this subject did not affect statistical values. **d** Chromosomal locations of DMRs. DMRs on autosomes are presented [65]. **e** Total number of DMRs and DMR-associated genes. **f** Venn diagrams of DMR-associated genes. MR methylated region, DMR differentially methylated region, PCA principal component analysis, PMI postmortem interval, CT control, BD bipolar disorder.

We performed promoter-wide DNA methylation analysis on NeuN-sorted neuronal (NeuN+) and nonneuronal (NeuN-) nuclear fractions derived from the PFC of patients with BD (*N* = 34) and controls (*N* = 35) (Table S1). DNA fragments containing densely methylated CpGs were enriched using the MBD2B/3L and analyzed with a promoter tiling array. PCA of the DNA methylation signature revealed a clear separation between neurons and nonneurons (Fig. 1a). We then assessed the effect of confounding factors on MRs and found that they did not significantly affect the total number of neuronal or nonneuronal MRs (Fig. 1a, c and Fig. S1). We then compared the total number of MRs per sample (Fig. 1b). Consistent with our previous report [16], the total number of MRs was significantly lower in neurons than in nonneurons within controls (*P* = 0.0006, in the Mann–Whitney test) and within patients (*P* = 4.75E−05). A significant decrease in the total number of MRs was also identified in both neurons and nonneurons of patients compared to controls (*P* = 0.0031 and *P* = 0.0318, respectively) (Fig. 1b). Less MRs in the patients was not dependent on the genomic context, such as repeat structure or segmental duplications (Fig. S2).

### Characterization of DMRs

**Fig. 2:**
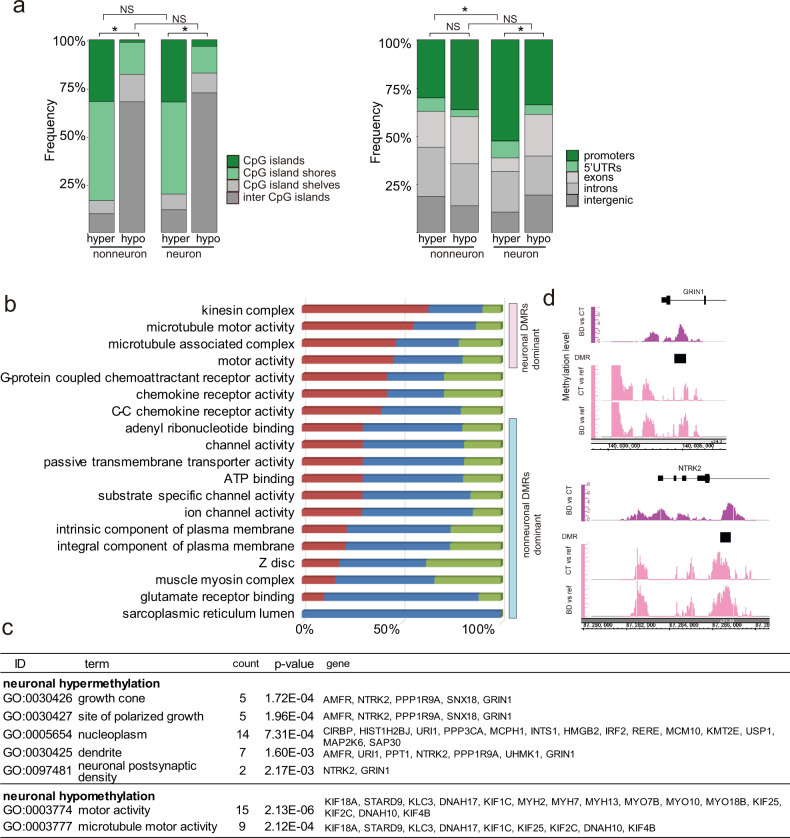
Characteristics of DMRs. **a** Genomic context of DMRs with regard to the CpG island and the gene structure. CpG island shore means up- and downstream 2 kb regions from the CpG island. CpG island shelf means up- and downstream 2 kb regions from the CpG island shore. An asterisk (*) indicates a significant difference in Fisher’s exact test (*P* < 0.05). NS not significant. **b** GO analysis of all DMR-associated genes. Significant GO terms were sorted based on the ascending order of the percentage of neuronal DMR-associated genes. Red, blue, and green colors indicate neuronal, nonneuronal, and common DMR-associated genes, respectively. See Table S4 for detailed results. **c** GO analysis of DMR-associated genes considering the cell type and direction of methylation changes. Only the results of neuronal DMR-associated genes are presented. See Table S5 for the results of nonneurons. **d** Example of neuronal hypermethylation at *GRIN1* and *NTRK2*. DMRs are denoted by black squares. The top panel shows DNA methylation levels determined by direct comparison of BD (*N* = 34) and CT (*N* = 35) samples. The peaks for the target group (BD) are shown. The bottom panels show the average DNA methylation levels determined by comparison of either CT (*N* = 35) or BD (*N* = 34) with references (whole-genome amplified, unmethylated human genome).

We then identified DMRs between BD patients and controls (Tables S2 and S3). The DMRs were uniformly distributed throughout the genome (Fig. 1d). Consistent with less MRs in BD, most DMRs showed hypomethylation (Fig. 1e). The overlaps between neurons and nonneurons at the gene level ranged from 23.4% to 44.1%. The rest showed cell-type-specific DNA methylation changes (Fig. 1f). We found that hypermethylated DMRs contained more CpG islands and shores than hypomethylated DMRs (Fig. 2a). We also found that neuronal hypermethylated DMRs contained more promoters and 5′-UTRs compared to neuronal hypomethylated and nonneuronal hypermethylated DMRs (Fig. 2a). We performed GO analysis using all the DMR-associated genes (Fig. 2b and Table S4). Each significantly enriched GO term was composed of neuronal and nonneuronal DMR-associated genes at different ratios. We found that kinesin complex-, microtubule-, and motor molecule-related genes dominantly included neuronal DMR-associated genes, whereas chemokine activity-related and inflammation-related genes were evenly enriched among both neuronal and nonneuronal DMR-associated genes. In contrast, ion channels and transporter-related terms mainly included nonneuronal DMR-associated genes. To further extract the cell-type-specific signature, we performed stratified GO analysis considering the cell type and direction of methylation change (Fig. 2c and Table S5). Strikingly, hypermethylated genes in neurons included genes related to the growth cone and dendrites (Fig. 2c), such as the NMDA NR1 subunit gene *GRIN1* and BDNF receptor gene *NTRK2* (Fig. 2d). Both genes have been the long-studied genes in psychiatric disorders, and their downregulation in the postmortem brains of BD patients has been established. On the other hand, kinesin complex- and microtubule motor activity-related genes were included in the hypomethylated genes of neurons (Fig. 2c and Table S5).

### Technical considerations of MRs and DMRs

We performed RRBS analysis in neurons and nonneurons of the selected subjects (Tables S1 and S6). Approximately 95% of the MRs detected in the array showed greater than 70% of the DNA methylation levels in RRBS, ensuring high sensitivity to the detection of methylated DNA (Fig. S3). A total of 999 DMRs contained at least one CpG site whose DNA methylation level could be determined by RRBS. Among them, 190 DMRs contained CpG(s) showing significant DNA methylation differences by RRBS. The average validation rates by RRBS were 16.4% for hypomethylation and 52.7% for hypermethylation (Table S7). The low rates of validation of DMRs largely came from the small number of samples used in RRBS compared to arrays. Hypermethylation changes were more supported by RRBS than hypomethylation changes. Because all arbitrarily chosen hypomethylated DMRs were successfully confirmed by independent qPCR (Fig. S4), we postulated the involvement of other epigenetic regulations such as hmC [26]. To explore the effect of hmC, we analyzed HMRs in neurons and nonneurons of two control samples using an anti-hmC antibody with the same array platform. We found that 12.1% of neuronal and 10.3% of nonneuronal MRs overlapped with neuronal and nonneuronal HMRs, respectively. We also found that overlaps with HMRs were significantly increased in DMRs; 17.8% of neuronal (Fisher’s exact test, *P* < 0.0001) and 13.7% of nonneuronal (*P* = 0.0045) DMRs in BD (Fig. S5, Tables S2 and S3). Based on the DMRs confirmed by RRBS, typical DNA methylation differences were estimated to range from 12.4 to 17.8% (Table S7).

### Assessment of the effect of mood stabilizers

**Fig. 3:**
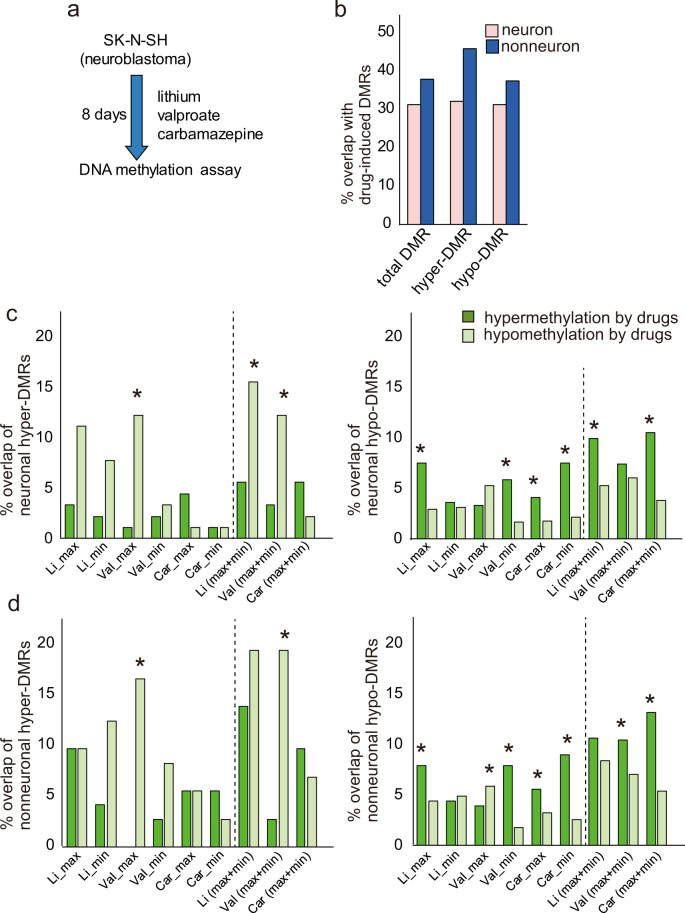
Effect of mood stabilizers on DMRs. **a** Experimental scheme. **b** % overlap with drug-induced DMRs. Detailed analysis of the overlap between either neuronal (**c**) or nonneuronal (**d**) DMRs and drug-induced DMRs. The abbreviations max, min, and (max + min) indicate maximum, minimum, and maximum or minimum therapeutic concentrations of mood stabilizers. An asterisk (*) indicates a significant difference in Fisher’s exact test (*P* < 0.05). Detailed information on overlapping DMRs is shown in Tables S2 and S3. Li lithium; Val valproate; Car carbamazepine.

We then assessed the effect of mood stabilizers on DNA methylation changes using a human neuroblastoma cell line. Cells cultured under the minimum and maximum therapeutic concentrations of three mood stabilizers for 8 days were retrieved, and their DNA methylation patterns were profiled with the same array platform (Fig. 3a). We examined the relationship between the DMRs in BD and those detected in cell culture. We found that 31.3% and 37.9% of the neuronal and nonneuronal DMRs, respectively, overlapped with DMRs detected in at least one cell culture condition (Fig. 3b, Tables S2 and S3). Regarding the direction of methylation changes in the DMRs, both directions showed a similar extent of overlap (Fig. 3b). Further analysis revealed that hypomethylated DMRs in BD showed a greater overlap with hypermethylated DMRs in cell culture and vice versa among both neuronal DMRs (Fig. 3c) and nonneuronal DMRs (Fig. 3d).

### Overlap analysis with GWAS in psychiatric disorders

**Fig. 4:**
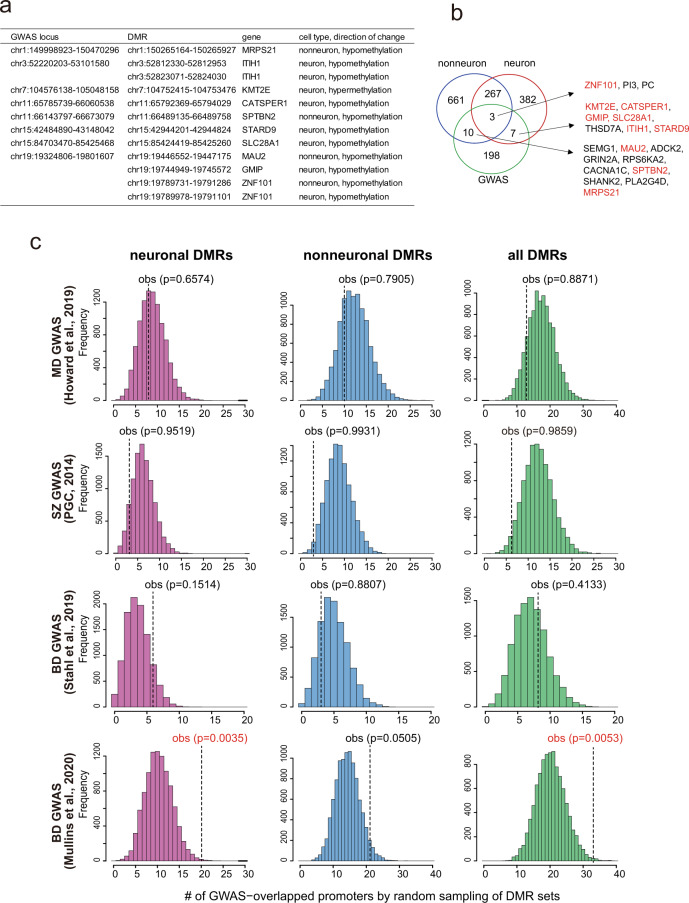
The overlap between GWAS loci and DMRs. **a** Overlap at the locus level. **b** Overlap at the gene level. Gene indicated in red shows that its DMR is included in the GWAS loci. Note that the total number of DMR-associated genes shows some inconsistencies with Fig. 1 due to the presence of genes having multiple DMRs of both directions of changes. GWAS loci and genes were retrieved from Stahl et al. [6]. **c** Enrichment test between the GWAS loci and DMRs by random sampling. GWAS loci were retrieved from the literature [6, 24, 27, 28]. Frequency was based on 10,000 random sampling of DMR sets. *P* value given in red indicates significant enrichment of DMRs.

We compared chromosomal loci identified by a GWAS in BD with the DMRs. Among the 30 loci identified by a GWAS in BD [6], 8 loci included a total of 12 DMRs (Fig. 4a). At the gene level, we also identified additional overlapped genes between GWAS and this study, including *CACNA1C*, *SHANK2*, and *GRIN2A* (Fig. 4b). We then compared the loci identified by other GWAS with the DMRs. We considered 102 loci in major depression (MD) [27], 108 loci in SZ [28], and 63 loci in BD [24]. We found that a total of 28, 14, and 63 DMRs overlapped with the loci reported in MD, SZ, and BD, respectively (Tables S2 and S3). To test if DMRs are significantly enriched in the GWAS loci, we performed the promoter-based Fisher’s exact test. Significant deviations were detected in the BD GWAS [24] and the SZ GWAS [28] loci, though directions of deviation seemed to be opposite (Table S8). To further test the enrichment, we also estimated *P* values from the probability distribution by 10,000 random sampling of DMR sets (Fig. 4c). Significant enrichment was detected in neuronal and all DMRs in the BD GWAS loci [24]. We also observed the depletion of DMRs in the SZ GWAS loci.

### qPCR of DNA methylation-related genes

**Fig. 5:**
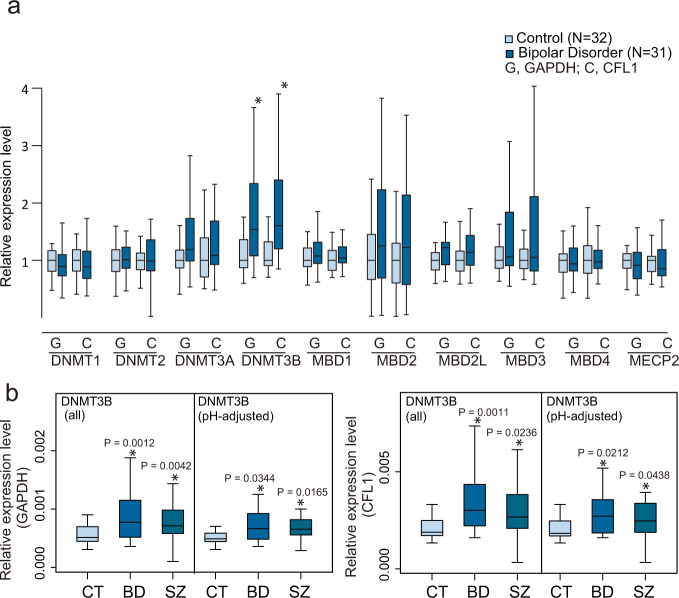
qPCR of DNA methylation-related genes. **a** Expression levels of DNA methylation-related genes. Expression levels were plotted relative to the average value of the control. Note that for some subjects, total RNA samples were not available. **b** The expression level of *DNMT3B*. The expression levels of two genes, GAPDH: G and CFL1: C, were used as internal controls. In the pH-adjusted analysis, samples for which the brain sample pH was below 6.4 were removed based on a previous study [30]. An asterisk (*) indicates a significant change in the Mann–Whitney test (*P* < 0.05).

To examine the genes involved in the DNA methylation changes in BD, we measured the gene expression levels of 4 DNA methyltransferases and 5 methyl-CpG binding domain-containing proteins by qPCR using bulk PFC samples (Fig. 5a). Among the measured genes, the expression of *DNMT3B* showed a significant increase in BD compared with controls (Fig. 5b). Specific and increased expression of *DNMT3B* was also found in the PFC of SZ patients (Fig. 5b), suggesting that *DNMT3B* is involved in altered DNA methylation in psychosis. Increased expression of *DNMT3B* in BD was also supported by the analysis of a previous DNA microarray dataset [29, 30] (Fig. S6).

## Discussion

We performed brain cell-type-specific DNA methylation analysis on the PFC of BD patients. Our analysis revealed a tendency toward decreased promoter methylation of both neurons and nonneurons and neuronal hypermethylation in some key genes important for neuronal function in BD.

We employed the enrichment of methylated DNA by MBD2B/3L followed by promoter tiling array analysis. Compared to the bisulfite sequencing (BS)-based method, this approach has limitations in the coverage of the genome and accuracy of the quantitative determination. However, taking advantage of the binding specificity of MBD2B, which does not bind hmC [26], we were able to enrich and analyze the MRs consisting of methylcytosine (mC) [31, 32]. Excluding hmC would be particularly important because hmC is enriched in neurons and cannot be discriminated from mC by the BS [17]. Therefore, the DMRs defined based on only the mC will be valuable for interpretation of epigenetic signatures in the brain. By performing promoter-wide analysis, we focused on the genomic regions directly important for gene expression regulation. Genome-wide analysis, such as MBD-Seq, would be useful for future studies to understand the entire role of epigenetic regulation in BD. Another limitation would be that due to the enrichment-based method, it cannot estimate the methylation level by calculating the ratio of methylated to unmethylated signals. However, the accuracy of quantification has also been proven in MBD-Seq by using appropriate reference set [31]. Although we could not adopt such correction in this study, we determined DMRs by independent duplicate assays, and we estimated that the DMRs showed substantial DNA methylation changes by RRBS analysis of the selected samples.

### DNA hypomethylation in BD

In control brains, we replicated the less neuronal MRs compared to nonneuronal MRs [16]. On the other hand, previous BS analyses by others reported higher methylated levels in neurons than in nonneurons [17, 21]. Discordance may come from differences in the data interpretation involving higher hmC levels in neurons and in the genomic region analyzed in this study, i.e., promoters in this study and the entire genomic region in other studies. The global tendency toward hypomethylation and gene-specific neuronal hypermethylation in BD was seemingly contradictory. Although the molecular mechanism and the relationship between these changes are unclear, such changes have also been observed in cancer cells [33]. Relevance to global DNA hypomethylation was also discussed in the Supplementary Discussion.

### DMR-associated genes

By GO analysis, we found that motor activity-related terms were enriched in DMR-associated genes. Most of them showed hypomethylated changes in neurons. Genes included the kinesin complex genes (*KIF*s and *KLC3*), myosin components (*MYH*s and *MYO*s), lipid transfer protein (*STARD9*), and dynein complexes (*DNAH17* and *DNAH10*), suggesting that motor molecules in neurons are widely dysregulated. Because neurons must transport synaptic vesicle precursors, neurotransmitter receptors, and mRNAs over long distances [34], dysregulation of motor activity affects diverse neuronal functions and the pathophysiology of psychiatric disorders. Interestingly, altered microtubule functions in neural stem and mature neural cells in BD have been recently reported [35].

We found hypermethylation of growth cone- and dendrite-related genes. Among them, *NTRK2* and *GRIN1* have been the long-studied genes in psychiatric disorders, and their downregulation in the postmortem brains of BD patients has been established. *NTRK2*, also known as TrkB, encodes a BDNF receptor and has been one of the prime targets in mood disorders. Decreased expression of *NTRK2* was repeatedly reported in postmortem brains of patients with psychiatric disorders [36–39] and animal models of depression [40–42]. The BDNF-NTRK2 signaling pathway is critical for the antidepressant effect of lamotorigine [43] and ketamine [44, 45] as well as the antimanic effect of lithium [46] in animal models. Genetic studies have revealed that *NTRK2* is associated with the treatment response to mood stabilizers in BD [47, 48] and suicidal behavior in mood disorders [49]. Interestingly, hypermethylation of the CpG island of the *NTRK2* promoter has been reported in suicide completers [50]. Because the identified region in this study was close to the CpG island, these methylation changes may be linked and contribute to the pathophysiology of psychiatric disorders.

NMDA receptors (NMDARs) mediate basic neuronal functions, and their dysfunction is closely linked to the pathophysiology of psychiatric disorders [51]. *GRIN1* (*NR1*) encodes an essential subunit of NMDAR, and its downregulation was reported in the postmortem brains of patients with psychiatric disorders [52]. *GRIN1* knockdown mice showed various behavioral alterations related to psychiatric disorders [53]. The involvement of altered DNA methylation of NMDAR genes, including *NR1*, which is associated with changes in expression and subunit composition, has been reported [54–56].

The discussion on other DMR-associated genes was described in the Supplementary Discussion.

### Effect of mood stabilizers

We observed that up to 37.9% of DMRs in BD overlapped with mood stabilizer-induced DMRs in cultured cells. Despite the simple cell culture model, these overlapping DMRs and opposite directions of changes between the postmortem brain and cell culture imply the pathophysiological importance of these DMRs. A similar opposite direction of DNA methylation changes related to mood stabilizers has been reported not only in a gene-specific manner [57] but also in systematic alterations in accelerated aging in BD [58]. Although the precise molecular mechanism remains unclear, mood stabilizers might normalize the epigenetic regulation in brain cells [9], leading to the amelioration of multiple DMRs between BD patients and controls.

### Comparison with GWAS results

At the chromosomal location level, among the DMRs overlapping between a BD GWAS [6] and this study, we regarded *KMT2E* and *SPTBN2* as particularly important. *KMT2E* encodes histone lysine methyltransferase 2E. The loss of function of histone lysine methyltransferases is involved in BD, SZ, and autism [59–61], and cell-type-specific alteration of histone lysine modification in postmortem brains and animal models of psychiatric disorders has been reported [62]. *SPTBN2*, also known as *SCA5*, regulates glutamate signaling by stabilizing EAAT4, and mutations in *SPTBN2* cause spinocerebellar ataxia type 5 [63]. At the gene level, several genes overlapped with GWAS, including the well-studied genes in BD [6] such as *CACNA1C*, *SHANK2*, and *GRIN2A*. They seemed to appear as candidates due to their long gene length.

### Enrichment and depletion of DMRs in the GWAS loci of psychiatric disorders

We developed the promoter-based Fisher’s exact test, where the number of DMR-overlapped promoter and that of DMR-nonoverlapped promoter were compared to the number of GWAS-overlapped promoter and that of GWAS-nonoverlapped promoter. *P* values were further evaluated based on the probability distribution estimated by the 10,000 random sampling of DMR sets. Strikingly, we found that neuronal DMRs were strongly enriched in the latest BD GWAS loci [24], but they were not enriched in the previous BD GWAS [6] or the MD GWAS [27] loci. This suggests that a larger scale of GWAS would be needed to detect a significant relationship. In contrast, DMRs were significantly depleted in the SZ GWAS loci [28]. Although careful considerations should be needed, this might be partly explained by the role of the SZ GWAS loci; they may play more roles in early neuronal development than in adulthood. The latest BD GWAS [24] showed the enrichment of GWAS signals such as calcium signaling genes and genes expressed in neurons. Enrichment of neuronal epigenetic alterations in the BD GWAS loci provides important insights into the molecular pathophysiology of BD.

### Overexpression of *DNMT3B* in BD and SZ

We found an increase in *DNMT3B* expression in both BD and SZ, implicating its possible role in psychosis. Whether patients with SZ show epigenetic changes similar to those of patients with BD needs to be studied. Increased expression of *DNMT3B* was recently reported in learned helplessness rats, supporting its role as a stress-inducible neuronal DNA methyltransferase [64].

## Conclusion

We observed cell-type-specific, pathophysiology-related DNA methylation changes in the PFC of patients with BD and identified increased expression of *DNMT3B* as a potential molecular mechanism. The present findings help understand the molecular pathophysiology of BD.

## Supplementary information

Supplementary Methods

Supplementary Discussion

Supplementary figures

Supplementary Tables S1 and S4 to S8

Supplementary Table S2

Supplementary Table S3

## Data Availability

The array data are available under accession GSE137921. The RRBS data are available under accession DRA008934.
