# Supplementary material for: Decreased DNA methylation at promoters and gene-specific neuronal hypermethylation in the prefrontal cortex of patients with bipolar disorder
Source: Mol Psychiatry. 2021 Apr 20;26(7):3407–18. doi: 10.1038/s41380-021-01079-0 (PMC8505249; doi:10.1038/s41380-021-01079-0)
Supplement: Supplementary file 1 — Supplementary Methods [file 41380_2021_1079_MOESM1_ESM.docx]

**Supplementary Methods**

**Cell culture**

Cell culture and drug conditions were as previously described^1^. In brief, cells of the human neuroblastoma cell line SK-N-SH (American Type Culture Collection) were cultured for 8 days in Eagle’s minimal essential medium containing 10% fetal bovine serum with one of three mood stabilizers. The medium was changed on days 2, 5, and 8. On day 9, the cells were retrieved. We used three mood stabilizers: lithium chloride (Sigma-Aldrich; St. Louis, MO, USA), valproic acid sodium salt (Sigma-Aldrich), and carbamazepine (Sigma-Aldrich). Valproate and lithium were directly dissolved into the medium. Carbamazepine was dissolved in dimethyl sulfoxide (DMSO) before it was added to the medium. The concentration of each drug was determined based on the therapeutic concentrations for BD. We prepared the following minimum and maximum therapeutic concentrations: valproate, 0.3 mM and 0.6 mM; lithium, 0.6 mM and 1.2 mM; and carbamazepine, 0.05 mM and 0.1 mM.

**Data analysis**

The number of MRs was compared using the Mann-Whitney test. Principal component analysis (PCA) of MRs based on Jaccard statistics was conducted using bedtools^2^. To assess the effect of confounding factors, continuous valuables including age, postmortem interval, brain pH, brain weight, age onset, duration of illness and lifetime antipsychotics (fluphenazine equivalents) were evaluated by Spearman correlation. The effect of categorical variables, including smoking (at the time of death), suicide status and presence of psychotic features were evaluated by Mann-Whitney test using either all available or patient samples.

The parameters used in the MAT were as follows: bandwidth, 300 bp; max gap, 300; min probe, 10; P-value, 1e-3. The MRs and DMRs on the sex chromosomes were excluded from this analysis. DMRs within multigene families, such as olfactory receptors and protocadherins, were removed from the analysis. Annotation was conducted using HOMER^3^. DMR-associated genes were additionally identified if the distance between the intergenic DMR and the nearest TSS was less than 5 kb. Genomic context of DMRs with regard to the CpG island and the gene structure was examined using the annotatr R package^4^. Gene Ontology (GO) analysis was performed with ToppGene^5^. The chromosomal location of the DMRs was visualized using CHARANGO software^6^.

To test if the overlap between DMRs and GWAS occurred randomly, we performed promoter-based Fisher’s exact test. Based on the annotation information of promoter tiling array (Affymetrix), promoters were assigned to either the DMR-overlapped promoter or the DMR-nonoverlapped promoter. Then, similarly, promoters were assigned to either the GWAS-overlapped promoter or the GWAS-nonoverlapped promoter. The total number of each category was compared by Fisher’s exact test. We also estimated P values from the probability distribution by 10,000 random sampling of DMR sets using R (v3.5.2).

**Reduced representation bisulfite sequencing (RRBS)**

RRBS was performed using the MethylSeq library construction, sequencing, and data analysis service (Zymo Research, Irvine, CA, USA). The total number of RRBS data points was 10 each for BD and controls. In brief, a total of 500 ng of genomic DNA was digested with Taq I and Msp I. DNA fragments were filled in, and A was added at the 3’-end, followed by adaptor ligation. After the bisulfite modification using the EZ DNA methylation-Direct kit (Zymo Research), preparative-scale PCR was performed. The size selection was performed on a 4% NuSieve 3:1 agarose gel. Library material was recovered using a Zymoclean Gel DNA recovery kit (Zymo Research). DNA sequencing was performed using an Illumina HiSeq2000. The data were analyzed using a Zymo Research proprietary pipeline (Zymo Research). We used CpG sites 1) with coverage ≥ 10 and 2) located within DMRs detected in the array analysis, which corresponded to 4,959 and 3,281 CpG sites for neuronal and nonneuronal DMRs, respectively. Statistical analyses were conducted by a one-tailed Student’s t-test.

**qPCR**

qPCR was performed according to a previous study^7^. A total of 1 µg of total RNA was used for cDNA synthesis by oligo (dT) and SuperScript II reverse transcriptase (Invitrogen). qPCR was performed using SYBR/GREEN I dye (Applied Biosystems, Foster City, CA, USA) with ABI PRISM 7900HT (Applied Biosystems). The comparative Ct method (Applied Biosystems) was employed for quantification. We used two internal control genes based on our previous analysis^7^. In addition to analyzing all the measured samples, we performed pH-adjusted analysis because the brain sample pH systematically affects the transcriptome^8^. Low pH samples (pH < 6.4) were omitted from the pH-adjusted analysis. The threshold was previously determined^9^. P < 0.05 in the Mann-Whitney test was considered significant. Primer pairs used in this study were as follows: MBD1, 5’- AGGAGGACAAGGAGGAGAACAA-3’ and 5’-GGCTGAAAATCTCCGTGATCAC-3’; MBD2, 5’-TCCAGGCAGAACCAATCCTTTC-3’ and 5’-AAAAGACATGGTCCCTGCCCT-3’; MBD2L, 5’-GTTTGGCTTAACACATCTCAACCC-3’ and 5’-GTACTCGCTCTTCCTGTTTCCTGA-3’; MBD3, 5’-GCTCCCTGTCAGAGTCAAAGCAC-3’ and 5’-GCACCAACCTCAGGAAGACGT-3’; MBD4, 5’-AATGGACACCTCCTCGGTCACC-3’ and 5’-CTTCCAAAGCACAGGTATTGCC-3’; DNMT1, 5’-CACTGCACGTGTTTGCTCC-3’ and 5’-ACCCGAGCTCAACCTGG-3’; DNMT2, 5'-TATGCGGTGACATGGATGAAC-3' and 5'-TCTCATCACCCCAATCAGAAAC-3'; DNMT3A, 5'-AACCTTCCCGGTATGAACAGGC-3' and 5'-TGCTGAACTTGGCTATCCTGCC-3'; DNMT3B, 5'-CCGTGACTGCAATAGAACCCTC-3' and 5'-AGAACTCAGCACACCCCTTCCT-3'; MECP2, 5'-GCCTCCTTTCCGTTTGATTTG-3' and 5'-CACATTGAGTAACAGTCCTGGTGA-3'. For each primer pair, amplification of the single product was confirmed by gel electrophoresis and by monitoring the dissociation curve.

**References**

1. Asai T, Bundo M, Sugawara H, Sunaga F, Ueda J, Tanaka G *et al.* Effect of mood stabilizers on DNA methylation in human neuroblastoma cells. *Int J Neuropsychopharmacol* 2013;**16(10):** 2285-2294.

2. Quinlan AR, Hall IM. BEDTools: a flexible suite of utilities for comparing genomic features. *Bioinformatics* 2010; **26**(6)**:** 841-842.

3. Heinz S, Benner C, Spann N, Bertolino E, Lin YC, Laslo P *et al.* Simple combinations of lineage-determining transcription factors prime cis-regulatory elements required for macrophage and B cell identities. *Mol Cell* 2010; **38**(4)**:** 576-589.

4. Cavalcante RG, Sartor MA. annotatr: genomic regions in context. *Bioinformatics* 2017; **33**(15)**:** 2381-2383.

5. Chen J, Bardes EE, Aronow BJ, Jegga AG. ToppGene Suite for gene list enrichment analysis and candidate gene prioritization. *Nucleic Acids Res* 2009; **37:** W305-311.

6. Nakachi Y, Ishii K, Bundo M, Masuda T, Iwamoto K. Use of the Illumina EPIC methylation array for epigenomic research in the crab-eating macaque (Macaca fascicularis). *Neuropsychopharmacol Rep* 2020; **40**(4)**:** 423-426.

7. Iwamoto K, Kakiuchi C, Bundo M, Ikeda K, Kato T. Molecular characterization of bipolar disorder by comparing gene expression profiles of postmortem brains of major mental disorders. *Mol Psychiatry* 2004; **9**(4)**:** 406-416.

8. Tomita H, Vawter MP, Walsh DM, Evans SJ, Choudary PV, Li J *et al.* Effect of agonal and postmortem factors on gene expression profile: quality control in microarray analyses of postmortem human brain. *Biol Psychiatry* 2004; **55**(4)**:** 346-352.

9. Iwamoto K, Bundo M, Kato T. Altered expression of mitochondria-related genes in postmortem brains of patients with bipolar disorder or schizophrenia, as revealed by large-scale DNA microarray analysis. *Hum Mol Genet* 2005; **14**(2)**:** 241-253.
