# Supplementary material for: Decreased DNA methylation at promoters and gene-specific neuronal hypermethylation in the prefrontal cortex of patients with bipolar disorder
Source: Mol Psychiatry. 2021 Apr 20;26(7):3407–18. doi: 10.1038/s41380-021-01079-0 (PMC8505249; doi:10.1038/s41380-021-01079-0)
Supplement: Supplementary file 2 — Supplementary Discussion [file 41380_2021_1079_MOESM2_ESM.docx]

**Supplementary Discussion**

**Effect of smoking**

Among the confounding factors, smoking has the profound effect on DNA methylation^1^. Although smoking status at time of death had no significant effects on neuronal and nonneuronal MRs, precise effect remains to be studied due to lack of information of smoking during life. We compared previous smoking MWAS data^1^, which reported 972 genomic regions, and DMRs identified in this study. We found only one genomic region overlapped with neuronal DMR and none overlapped with nonneuronal DMRs. An overlapped neuronal DMR located in the *TERT* gene (chr5:1268069-1269345) and showed hypomethylation in BD. Although the role of *TERT* in neurons*,* which encodes telomerase reverse transcriptase, was potentially interesting, we concluded that the effect of smoking was negligible in this study.

**Global DNA hypomethylation**

Global DNA hypomethylation is frequently reported in blood cells of psychiatric disorders, including BD^2-4^. We also reported global DNA hypomethylation in BD and SZ in blood cells, and hypomethylation was associated with the serum level of the methyl-group donor betaine (*N,N,N*-trimethylglycine)^5^. Whether hypomethylation in the PFC of BD patients is accompanied by similar metabolite changes remains unclear. Systematic DNA methylation changes across different tissues have been reported during normal aging, enabling the establishment of the epigenetic clock^6^. Interestingly, BD patients showed accelerated aging in blood and brain tissues^7, 8^. It would be interesting to pursue the relationship between promoter-wide hypomethylation and accelerated aging in BD.

**Interpretation of DMR-associated genes**

We observed common DNA methylation changes of chemokine activity- and inflammation-related genes such as *CCR1*, *CCR3*, *CCR5*, *CCR6*, *CXCR1*, and *CX3CR1*. Chemokines and their receptors have neurotransmitter-like effects and have roles in the diverse neurological processes such as neuron-glia interaction and neurogenesis. They are known to be involved in the pathophysiology of psychiatric disorders^9^.

Other characteristic findings include a wide range of DNA methylation changes in ion channel- and transporter-related genes in nonneurons. For example, they include the calcium channels (*CACNA1C, CACNA2D4, CACNB2,* and *CACNG8*), glutamate receptors *(GRIK2, GRIN2A,* and *GRM8*), GABA receptors (*GABRA5, GABRG3, GABRG3,* and *GABRP*), and potassium channels (*KCNA1, KCNA4, KCNA7, KCNAB2, KCNAB2, KCND3, KCND3, KCNE1,* and *KCNG2*). A recent GWAS showed enrichment of GWAS signals in calcium signaling genes and genes expressed in neurons^10^. The current finding of DNA methylation changes in ion channels, including calcium channels, sheds light on the potential roles of these channels in nonneurons, such as oligodendrocytes, microglia, and astrocytes, in BD. Further analysis of the specific nonneuronal cell population will be important.

**References**

1. Zeilinger S, Kuhnel B, Klopp N, Baurecht H, Kleinschmidt A, Gieger C *et al.* Tobacco smoking leads to extensive genome-wide changes in DNA methylation. *PLoS One* 2013; **8**(5)**:** e63812.

2. Melas PA, Rogdaki M, Osby U, Schalling M, Lavebratt C, Ekstrom TJ. Epigenetic aberrations in leukocytes of patients with schizophrenia: association of global DNA methylation with antipsychotic drug treatment and disease onset. *FASEB J* 2012; **26**(6)**:** 2712-2718.

3. Huzayyin AA, Andreazza AC, Turecki G, Cruceanu C, Rouleau GA, Alda M *et al.* Decreased global methylation in patients with bipolar disorder who respond to lithium. *The international journal of neuropsychopharmacology / official scientific journal of the Collegium Internationale Neuropsychopharmacologicum* 2014; **17**(4)**:** 561-569.

4. Li S, Yang Q, Hou Y, Jiang T, Zong L, Wang Z *et al.* Hypomethylation of LINE-1 elements in schizophrenia and bipolar disorder. *J Psychiatr Res* 2018; **107:** 68-72.

5. Murata Y, Ikegame T, Koike S, Saito T, Ikeda M, Sasaki T *et al.* Global DNA hypomethylation and its correlation to the betaine level in peripheral blood of patients with schizophrenia. *Prog Neuropsychopharmacol Biol Psychiatry* 2020; **99:** 109855.

6. Horvath S. DNA methylation age of human tissues and cell types. *Genome Biol* 2013; **14**(10)**:** R115.

7. Fries GR, Zamzow MJ, Andrews T, Pink O, Scaini G, Quevedo J. Accelerated aging in bipolar disorder: A comprehensive review of molecular findings and their clinical implications. *Neuroscience and biobehavioral reviews* 2020; **112:** 107-116.

8. Fries GR, Zamzow MJ, Colpo GD, Monroy-Jaramillo N, Quevedo J, Arnold JG *et al.* The anti-aging effects of lithium in lymphoblastoid cell lines from patients with bipolar disorder and controls. *J Psychiatr Res* 2020; **128:** 38-42.

9. Stuart MJ, Baune BT. Chemokines and chemokine receptors in mood disorders, schizophrenia, and cognitive impairment: a systematic review of biomarker studies. *Neuroscience and biobehavioral reviews* 2014; **42:** 93-115.

10. Mullins N, Forstner AJ, O'Connell KS, Coombes B, Coleman JRI, Qiao Z *et al.* Genome-wide association study of over 40,000 bipolar disorder cases provides novel biological insights. *medRxiv* 2020.
