# Supplementary material for: Decreased DNA methylation at promoters and gene-specific neuronal hypermethylation in the prefrontal cortex of patients with bipolar disorder
Source: Mol Psychiatry. 2021 Apr 20;26(7):3407–18. doi: 10.1038/s41380-021-01079-0 (PMC8505249; doi:10.1038/s41380-021-01079-0)
Supplement: Supplementary file 3 — Supplementary figures [file 41380_2021_1079_MOESM3_ESM.docx]

**Supplementary Figures**


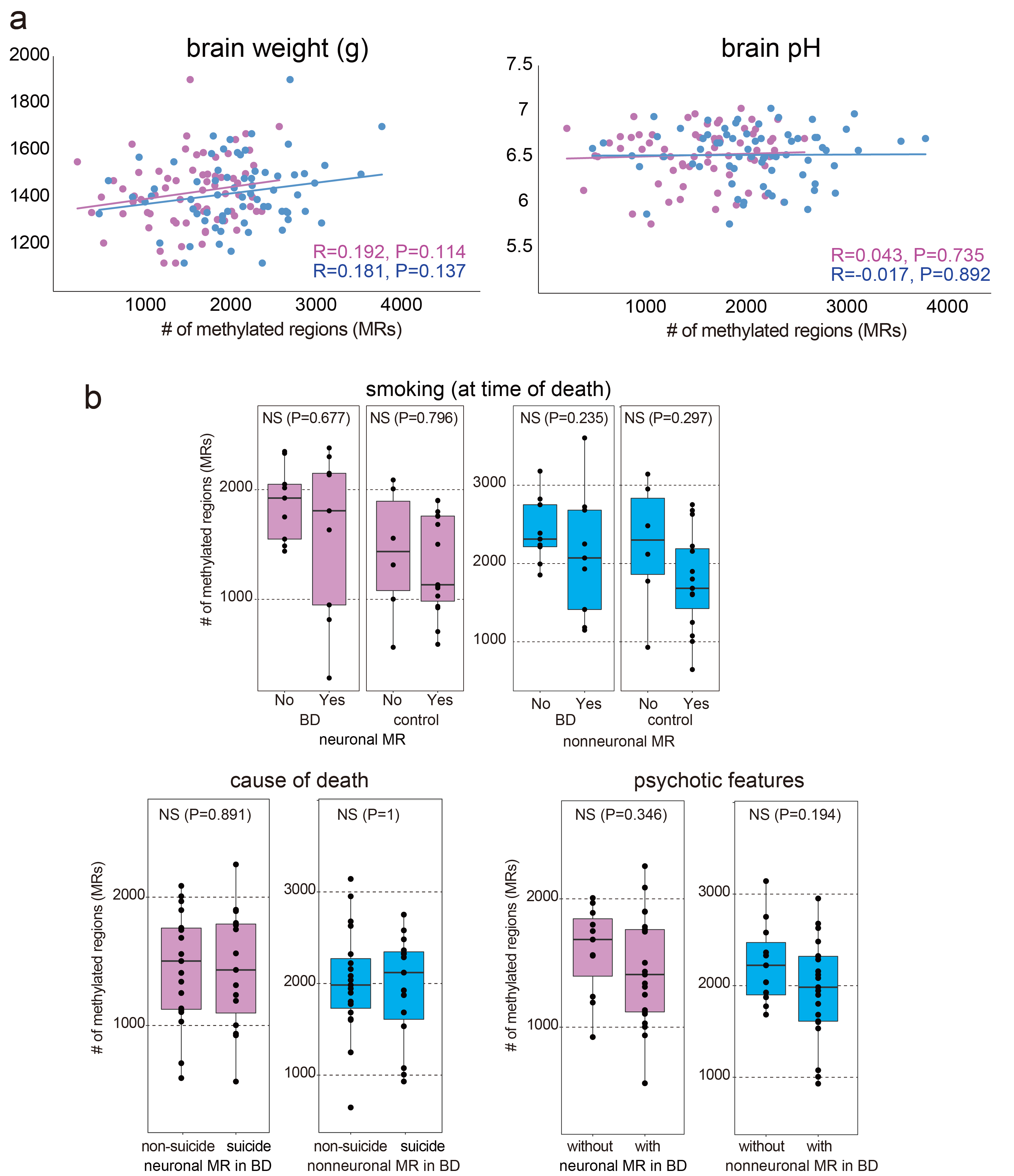


**Figure S1. Effect of confounding factors on MRs.** a. Effect of brain weight and brain pH on MRs. Spearman's rank correlation coefficient and P value are given. Pink and blue colors indicate neurons and nonneurons, respectively. b. Effect of smoking status at the time of death, cause of death, and psychotic features on MRs. P values by Mann-Whitney test are given. MR, methylated region; BD, bipolar disorder; NS, not significant.


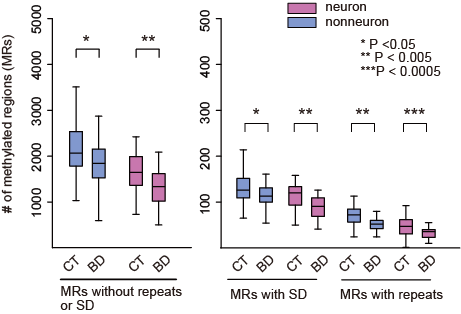


**Figure S2. Decreased number of MRs in BD.** Left panel: comparison of the average number of MRs excluding the MRs that overlapped with repetitive sequences or segmental duplications. Right panel: comparison of the average number of MRs that overlapped with repetitive sequences or segmental duplication. Note that the decreased number of MRs in BD was not influenced by the cell type or genomic context. CT, control; BD, bipolar disorder; MR, methylated region; SD, segmental duplication.


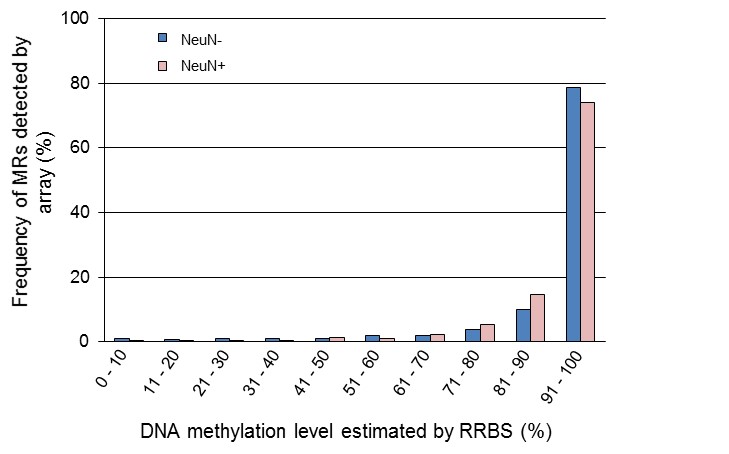


**Figure S3. MRs identified by array analysis were efficiently detected by RRBS**. For each MR detected by array, the average DNA methylation level estimated by RRBS was calculated. Approximately 95% of MRs were estimated to have greater than 70% of the DNA methylation level in both NeuN+ and NeuN- samples. DNA methylation levels were calculated using the CpGs with coverage greater than 10. A representative result (from one subject) is shown here.


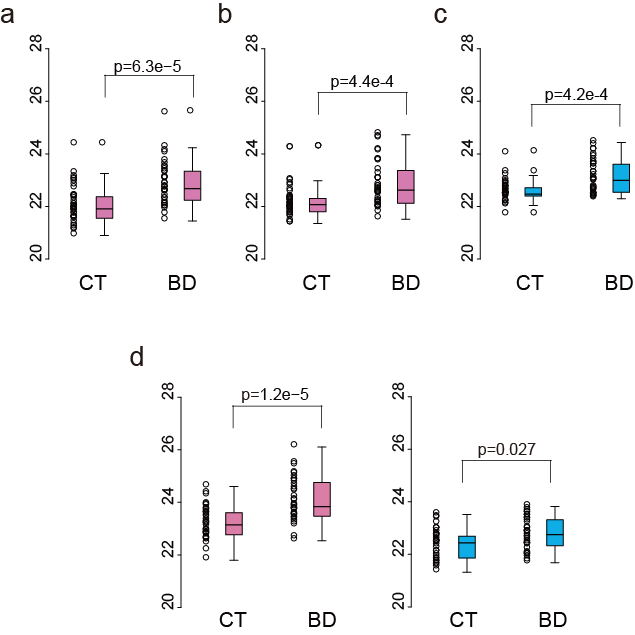


**Figure S4. qPCR validation of hypomethylated regions in BD.** a. Neuronal hypomethylation at chr8:6478908-6480044 in *MCPH1*. b. Neuronal hypomethylation at chr4:57459500-57460123 in *THEGL*. c. Nonneuronal hypomethylation at chr19:49932808-49933573 in *SLC17A7*. d. Neuronal and nonneuronal hypomethylation at chr2:136593910-136594677 in *LCT*. For qPCR, aliquots of eluted methylated DNA were used for quantification. The CT value of each sample was plotted. P values were obtained by the Mann-Whitney test.


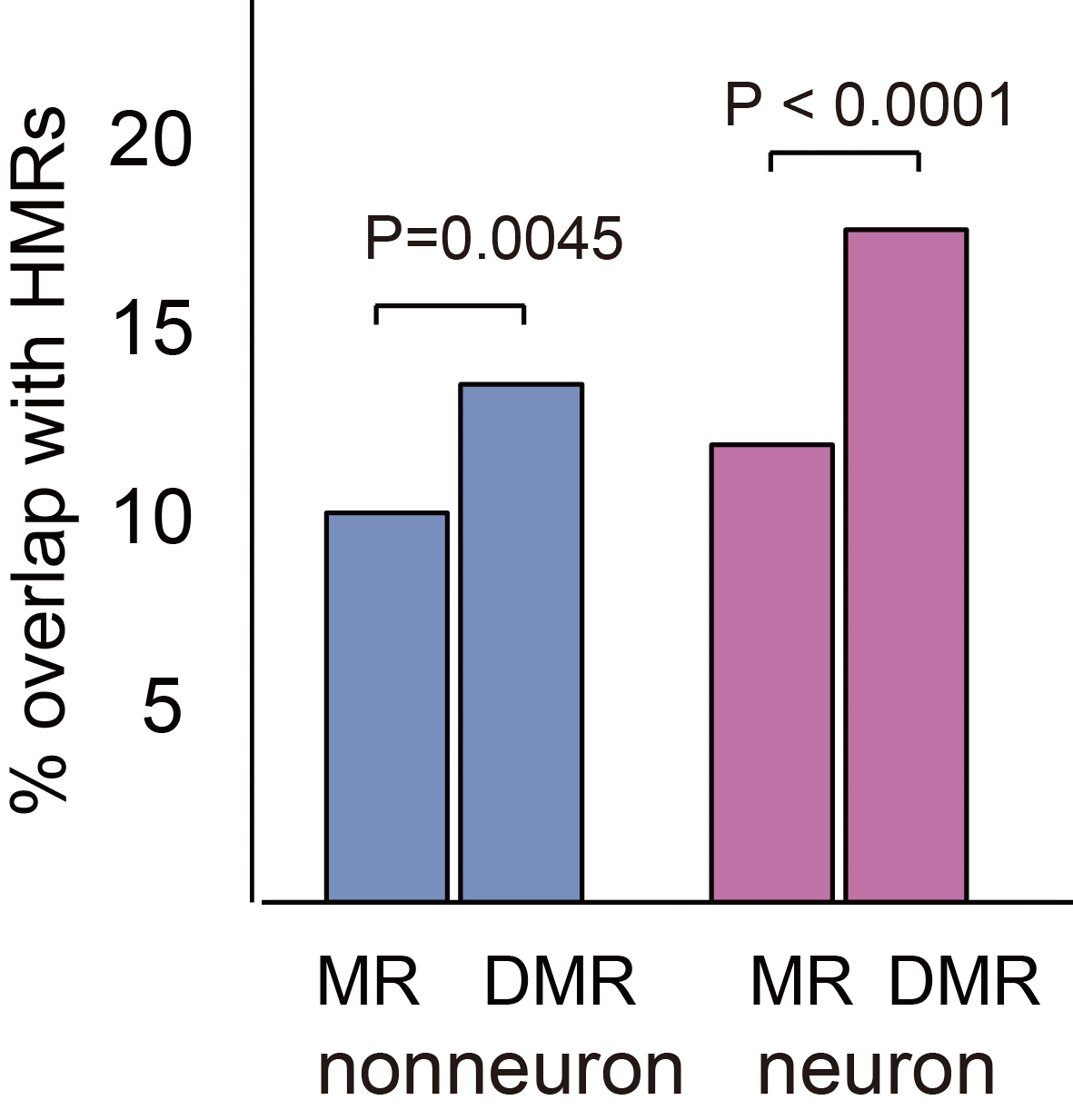


**Figure S5. Overlap between MR or DMR and HMRs.** HMRs are determined in two control subjects. MR, methylated region; DMR, differentially methylated region. HMRs, hydroxymethylated regions. P values were obtained by Fisher’s exact test.


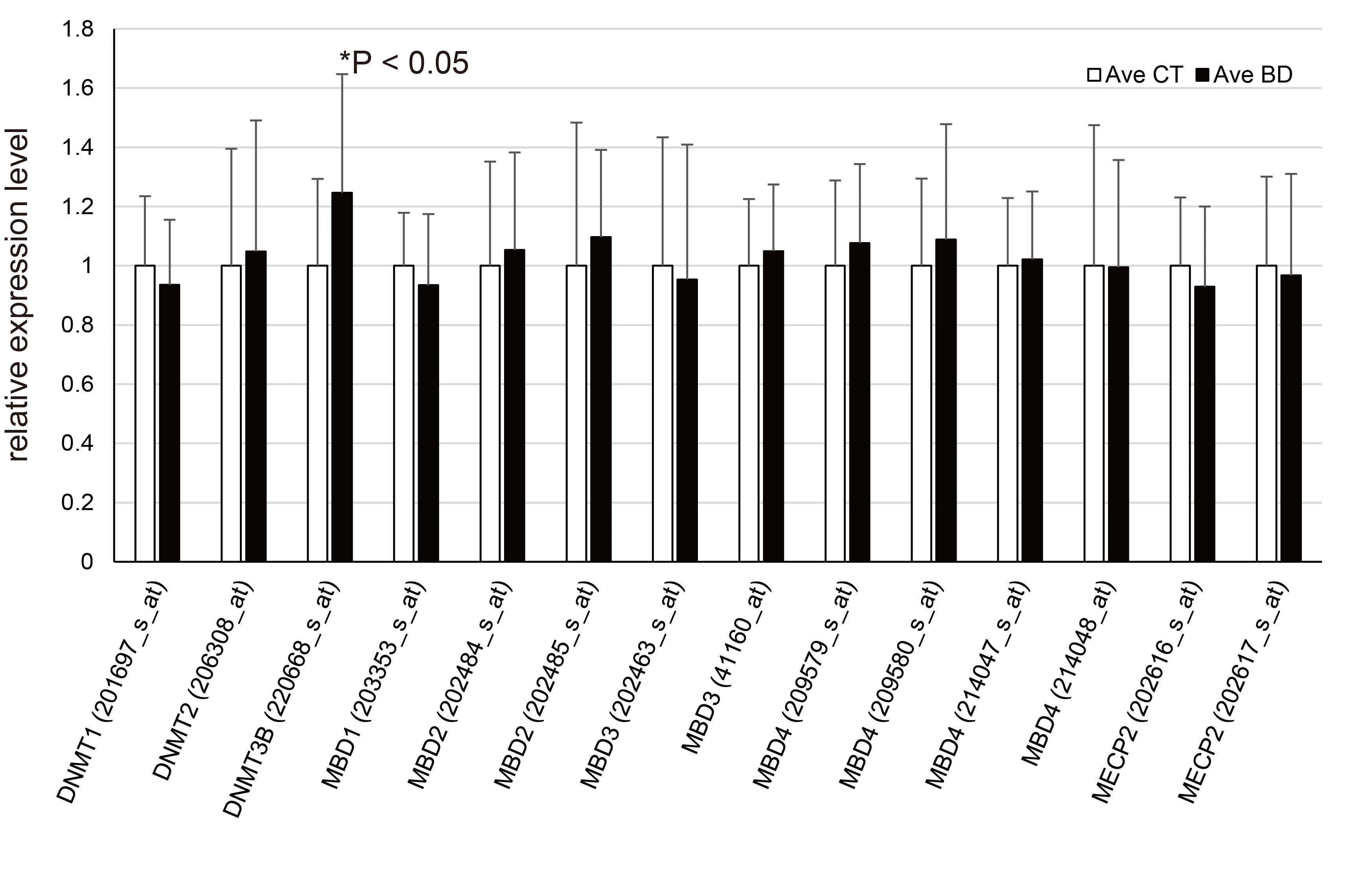


**Figure S6. Expression levels of DNA methylation-related genes by microarray analysis.** By utilizing the previously performed gene expression analysis with HGU133A microarrays (Iwamoto et al., Hum Mol Genet 2015), expression levels of DNA methylation-related genes in CT (N = 34) and BD (N = 33) were examined. Among the 10 genes tested by qPCR, we found that 14 probes for 8 genes were found to be expressed in the prefrontal cortex samples. Expression values were obtained by MAS5 normalization. Relative expression level was calculated by dividing average expression value of CT. Bar indicates the standard deviation. P values were obtained by the Mann-Whitney test.
