# Supplementary material for: Decreased DNA methylation at promoters and gene-specific neuronal hypermethylation in the prefrontal cortex of patients with bipolar disorder
Source: Mol Psychiatry. 2021 Apr 20;26(7):3407–18. doi: 10.1038/s41380-021-01079-0 (PMC8505249; doi:10.1038/s41380-021-01079-0)
Supplement: Supplementary file 4 — Supplementary Tables S1 and S4 to S8 [file 41380_2021_1079_MOESM4_ESM.pdf]

**Table S1. Summary of demographic variables of postmortem brains**

| | CT (array, qPCR, qRT-PCR) | BD (array, qPCR, qRT-PCR) | SZ (qRT-PCR) |
|---|---|---|---|
| # subjects | 35 | 34 | 35 |
| Age | 44.2 (7.6) | 45.4 (10.7) | 42.6 (8.5) |
| Sex (M:F) | 26:9 | 16:18 | 26:9 |
| PMI (hours) | 29.4 (12.9) | 37.6 (18.4) | 31.4 (15.5) |
| sample pH | 6.6 (0.3) | 6.4 (0.3) | 6.5 (0.2) |
| | CT (RRBS) | BD (RRBS) | |
| # subjects | 5 | 5 | |
| Age | 42.4 (11.3) | 43.8 (13.1) | |
| Sex (M:F) | 3:2 | 3:2 | |
| PMI (hours) | 27.4 (13.1) | 31.6 (17.7) | |
| sample pH | 6.62 (0.13) | 6.79 (0.15) | |

Average (SD) is given in age and PMI, and sample pH. In array and RRBS analyses, both NeuN+ and NeuN- samples were independently analyzed in each subject. Samples used for RRBS were selected from those used for array analysis. CT, control; BD, bipolar disorder; SZ, schizophrenia; PMI, postmortem interval; RRBS, reduced representation bisulfite sequencing.

**Table S4. Gene ontology analysis of DMR-associated genes using ToppGene.**

| ID | Name | Hit Count in Query | Hit Count in Genome | p-value | FDR B&H |
|---|---|---|---|---|---|
| GO:0003774 | motor activity | 26 | 130 | 9.03E-08 | 1.31E-04 |
| GO:0003777 | microtubule motor activity | 16 | 77 | 1.60E-05 | 1.16E-02 |
| GO:0022803 | passive transmembrane transporter activity | 48 | 432 | 6.21E-05 | 2.25E-02 |
| GO:0015267 | channel activity | 48 | 432 | 6.21E-05 | 2.25E-02 |
| GO:0022838 | substrate-specific channel activity | 45 | 408 | 1.24E-04 | 3.58E-02 |
| GO:0005887 | integral component of plasma membrane | 117 | 1364 | 1.37E-04 | 9.37E-02 |
| GO:0005216 | ion channel activity | 42 | 397 | 4.94E-04 | 9.58E-02 |
| GO:0016493 | C-C chemokine receptor activity | 5 | 12 | 4.96E-04 | 9.58E-02 |
| GO:0004950 | chemokine receptor activity | 7 | 25 | 6.14E-04 | 9.58E-02 |
| GO:0001637 | G-protein coupled chemoattractant receptor activity | 7 | 25 | 6.14E-04 | 9.58E-02 |
| GO:0035254 | glutamate receptor binding | 9 | 41 | 7.51E-04 | 9.58E-02 |
| GO:0032559 | adenyl ribonucleotide binding | 124 | 1517 | 7.63E-04 | 9.58E-02 |
| GO:0005524 | ATP binding | 121 | 1476 | 7.94E-04 | 9.58E-02 |
| GO:0005871 | kinesin complex | 11 | 53 | 3.19E-04 | 9.68E-02 |
| GO:0031226 | intrinsic component of plasma membrane | 118 | 1418 | 4.24E-04 | 9.68E-02 |
| GO:0005875 | microtubule associated complex | 19 | 138 | 8.04E-04 | 9.92E-02 |
| GO:0033018 | sarcoplasmic reticulum lumen | 4 | 8 | 8.18E-04 | 9.92E-02 |
| GO:0005859 | muscle myosin complex | 6 | 20 | 9.84E-04 | 9.92E-02 |
| GO:0030018 | Z disc | 16 | 109 | 1.01E-03 | 9.92E-02 |

DMR, differentially methylated region.

**Table S5. Detailed gene ontology analysis**

| ID | Name | Hit Count in Query | Hit Count in Genome | p-value | FDR B&H | gene |
|---|---|---|---|---|---|---|
| **neuronal hypermethylation** | | | | | | |
| GO:0030426 | growth cone | 5 | 142 | 1.72E-04 | 2.11E-02 | AMFR, NTRK2, PPP1R9A, SNX18, GRIN1 |
| GO:0030427 | site of polarized growth | 5 | 146 | 1.96E-04 | 2.11E-02 | AMFR, NTRK2, PPP1R9A, SNX18, GRIN1 |
| GO:0005654 | nucleoplasm | 14 | 1465 | 7.31E-04 | 5.26E-02 | CIRBP, HIST1H2BJ, URI1, PPP3CA, MCPH1, INTS1, HMGB2, IRF2, RERE, MCM10, KMT2E, USP1, MAP2K6, SAP30 |
| GO:0030425 | dendrite | 7 | 469 | 1.60E-03 | 8.66E-02 | AMFR, URI1, PPT1, NTRK2, PPP1R9A, UHMK1, GRIN1 |
| GO:0097481 | neuronal postsynaptic density | 2 | 19 | 2.17E-03 | 9.36E-02 | NTRK2, GRIN1 |
| **neuronal hypomethylation** | | | | | | |
| GO:0003774 | motor activity | 15 | 130 | 2.13E-06 | 1.93E-03 | KIF18A, STARD9, KLC3, DNAH17, KIF1C, MYH2, MYH7, MYH13, MYO7B, MYO10, MYO18B, KIF25, KIF2C, DNAH10, KIF4B |
| GO:0003777 | microtubule motor activity | 9 | 77 | 2.12E-04 | 9.59E-02 | KIF18A, STARD9, KLC3, DNAH17, KIF1C, KIF25, KIF2C, DNAH10, KIF4B |
| **nonneuronal hypermethylation** | | | | | | |
| GO:0006486 | protein glycosylation | 6 | 279 | 8.08E-05 | 2.64E-02 | LMF1, DAD1, MUC19, GNPTG, MUC3A, MGAT4C |
| GO:0043413 | macromolecule glycosylation | 6 | 279 | 8.08E-05 | 2.64E-02 | LMF1, DAD1, MUC19, GNPTG, MUC3A, MGAT4C |
| GO:0044723 | single-organism carbohydrate metabolic process | 9 | 723 | 8.70E-05 | 2.64E-02 | PPP1R3D, LMF1, P2RY1, DAD1, MUC19, CHST1, GNPTG, MUC3A, MGAT4C |
| GO:0070085 | glycosylation | 6 | 290 | 1.00E-04 | 2.64E-02 | LMF1, DAD1, MUC19, GNPTG, MUC3A, MGAT4C |
| **nonneuronal hypomethylation** | | | | | | |
| GO:0005887 | integral component of plasma membrane | 87 | 1364 | 6.19E-05 | 3.84E-02 | MAS1, MC2R, RXFP3, MEP1A, ESYT2, ADRB2, KIAA1324, SCNN1A, FGFR4, KCNT1, VN1R4, GPR65, ANPEP, ITGA8, AQP7, SLC6A4, PDPN, SLC6A11, SLC6A13, TNFSF11, SHANK2, TENM3, SLC16A1, SLC34A1, SLCO2A1, MTNR1A, KCNK5, GABRA5, GPRC5C, TNFRSF21, CD300C, CEACAM1, SLC52A3, NPFFR2, GJA8, SLC38A3, GPR4, CACNB2, NPR3, NPY2R, SLC11A2, GPR39, FFAR2, ROR1, GRIN2A, SLC13A2, GRM8, OPRM1, SLC7A9, SLC16A5, CD70, TPO, NRG1, CHUK, SLC39A6, CCR1, CCR3, CCR5, FLRT3, CNGA1, PLXNA2, TRPV1, COL17A1, HTR3A, PODXL2, CSF3R, CX3CR1, TRABD2B, TNFRSF9, ITGA4, PTAFR, ITGAE, KCNA1, SLC13A4, KCNA4, KCNA7, PTPRN2, KCND3, KCNE1, KIR3DL1, TUSC3, LCT, CACNG8, GPR68, HRH4, LYN, TACSTD2 |
| GO:0033018 | sarcoplasmic reticulum lumen | 4 | 8 | 1.90E-04 | 4.81E-02 | SRL, ASPH, CASQ2, HRC |
| GO:0031226 | intrinsic component of plasma membrane | 87 | 1418 | 2.33E-04 | 4.81E-02 | MAS1, MC2R, RXFP3, MEP1A, ESYT2, ADRB2, KIAA1324, SCNN1A, FGFR4, KCNT1, VN1R4, GPR65, ANPEP, ITGA8, AQP7, SLC6A4, PDPN, SLC6A11, SLC6A13, TNFSF11, SHANK2, TENM3, SLC16A1, SLC34A1, SLCO2A1, MTNR1A, KCNK5, GABRA5, GPRC5C, TNFRSF21, CD300C, CEACAM1, SLC52A3, NPFFR2, GJA8, SLC38A3, GPR4, CACNB2, NPR3, NPY2R, SLC11A2, GPR39, FFAR2, ROR1, GRIN2A, SLC13A2, GRM8, OPRM1, SLC7A9, SLC16A5, CD70, TPO, NRG1, CHUK, SLC39A6, CCR1, CCR3, CCR5, FLRT3, CNGA1, PLXNA2, TRPV1, COL17A1, HTR3A, PODXL2, CSF3R, CX3CR1, TRABD2B, TNFRSF9, ITGA4, PTAFR, ITGAE, KCNA1, SLC13A4, KCNA4, KCNA7, PTPRN2, KCND3, KCNE1, KIR3DL1, TUSC3, LCT, CACNG8, GPR68, HRH4, LYN, TACSTD2 |

**Table S6. Summary statistics of RRBS**

| | Sample Name | Cell type | Total Read # | Mapped Read # | Mapping Ratio | Unique CpG | BS Conv. Rate |
|---|---|---|---|---|---|---|---|
| CT | CT1 | NeuN+ | 25,609,162 | 14,038,544 | 54.82% | 8,671,005 | 97.75% |
| | CT1 | NeuN- | 34,561,870 | 19,815,410 | 57.33% | 9,287,088 | 98.63% |
| | CT2 | NeuN+ | 25,889,320 | 14,206,635 | 54.87% | 8,774,939 | 97.48% |
| | CT2 | NeuN- | 24,364,626 | 13,284,960 | 54.53% | 8,403,867 | 99.05% |
| | CT3 | NeuN+ | 24,399,994 | 13,830,671 | 56.68% | 8,513,356 | 97.95% |
| | CT3 | NeuN- | 20,554,802 | 11,902,338 | 57.91% | 8,278,046 | 98.97% |
| | CT4 | NeuN+ | 27,684,153 | 14,579,873 | 52.67% | 8,734,260 | 97.90% |
| | CT4 | NeuN- | 30,264,145 | 15,569,667 | 51.45% | 8,722,780 | 98.66% |
| | CT5 | NeuN+ | 28,271,910 | 14,842,290 | 52.50% | 8,800,643 | 97.80% |
| | CT5 | NeuN- | 28,631,102 | 15,379,802 | 53.72% | 8,719,988 | 98.85% |
| BD | BD1 | NeuN+ | 28,465,773 | 14,222,031 | 49.96% | 8,683,633 | 97.92% |
| | BD1 | NeuN- | 24,881,248 | 13,582,015 | 54.59% | 8,481,104 | 98.91% |
| | BD2 | NeuN+ | 26,956,772 | 14,636,382 | 54.30% | 8,675,243 | 98.03% |
| | BD2 | NeuN- | 25,611,650 | 13,464,313 | 52.57% | 8,570,716 | 99.14% |
| | BD3 | NeuN+ | 34,404,962 | 16,832,308 | 48.92% | 8,342,360 | 98.00% |
| | BD3 | NeuN- | 24,522,187 | 12,860,640 | 52.44% | 8,413,745 | 98.97% |
| | BD4 | NeuN+ | 25,858,959 | 14,103,985 | 54.54% | 8,604,493 | 97.92% |
| | BD4 | NeuN- | 23,035,051 | 12,697,691 | 55.12% | 8,579,386 | 98.94% |
| | BD5 | NeuN+ | 28,734,711 | 13,320,990 | 46.36% | 8,737,260 | 97.97% |
| | BD5 | NeuN- | 27,051,126 | 10,844,672 | 40.09% | 7,989,470 | 99.01% |

RRBS, reduced representation bisulfite sequencing; BS Conv. Rate, bisulfite modification-conversion rate; CT, control; BD, bipolar disorder.

**Table S7. Validation of DMRs by RRBS**

| | | # of tested DMRs | # of confirmed DMRs | DNA methylation differences of confirmed DMRs |
|---|---|---|---|---|
| neuron | hypomethylation | 406 | 73 (18.0%) | -13.1 ± 8.0 |
| | hypermethylation | 46 | 24 (52.2%) | 12.4 ± 7.1 |
| nonneuron | hypomethylation | 515 | 76 (14.8%) | -17.8 ± 8.3 |
| | hypermethylation | 32 | 17 (53.1%) | 17.4 ± 8.4 |

RRBS, reduced representation bisulfite sequencing; DMR, differentially methylated region.

**Table S8. Promoter-based Fisher's exact test.**

| | MD GWAS (Howard et al. 2019) | SZ GWAS (PGC, 2014) | BD GWAS (Stahl et al. 2019) | BD GWAS (Mullins et al. 2020) |
|---|---|---|---|---|
| neuronal DMR | 0.865 | 0.223 | 0.185 | 0.006 |
| nonneuronal DMR | 0.561 | 0.037 | 0.493 | 0.103 |
| all DMR | 0.268 | 0.056 | 0.701 | 0.008 |

P values are obtained by Fisher's exact test. MD, major depression; SZ, schizophrenia; BD, bipolar disorder; GWAS, genome-wide association study; DMRs, differentially methylated region.
